# Enhanced response of titanium doped iron(ii) oxalate under electric field†

**DOI:** 10.1039/d2ra05608a

**Published:** 2022-11-08

**Authors:** Chunde Li, Hua Wei, Xueyan Hu, Zhaoxian Chen, Xin Xie, Guo Chen, Anping Liu, Yingzhou Huang, Weijia Wen

**Affiliations:** Chongqing Key Laboratory of Soft Condensed Matter Physics and Smart Materials, College of Physics, Chongqing University Chongqing 401331 China huawei.hw@cqu.edu.cn; State Key Laboratory of Mechanical Transmissions, Chongqing University Chongqing 400044 China; Department of Physics, The Hong Kong University of Science and Technology Clear Water Bay Kowloon Hong Kong China

## Abstract

Electrorheological (ER) fluid, containing polarized particles within an insulating liquid, represents a smart material, the mechanical properties of which can be altered mainly by an electric field. In this work, ER fluids based on cauliflower iron(ii) oxalate doped titanium particles show excellent rheological and wetting properties by the sample co-precipitation method. The morphology of the particles is observed by SEM and the molecular structure within the particles is obtained *via* XRD and FTIR. The distribution of elements within the particles is obtained by EDS. Owing to a lower current density than pure iron(ii) oxalate, the SEM and optical images show an obvious chain-like structure within the ER fluids with 2 wt% and 5 wt%, respectively, under 2 kV mm^−1^. Then, the rheological properties of these ER fluids are tested up to 3 kV mm^−1^ and the results show a gratifying property of resisting shear with different shear rates (0.1–100 s^−1^), which is attributed to the appearance of a stable chain-like structure. At the same time, the ER efficiency and the switching performance are obtained and the static yield stress fits the relevant electric field strength well. Ultimately, an excellent sedimentation ratio is obtained from 0 h to 600 h.

## Abstract 2

Electrorheological (ER) fluid, containing polarized particles within an insulating liquid, represents a smart material, the mechanical properties of which can be altered mainly by an electric field.

## Graphical abstract

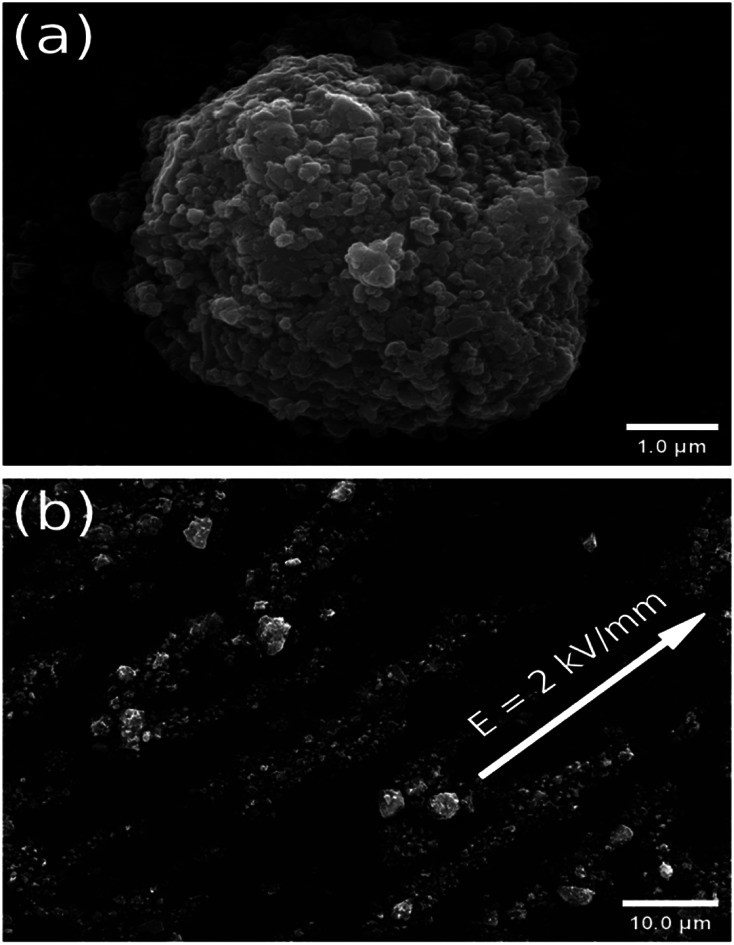

## Introduction

Recently, the development of smart materials, which can be controlled by an electric field, magnetic field, light, temperature, *etc.*,^1–3^ has attracted increasingly more attention. Electrorheological (ER) fluid, which exhibits flow behavior and rheological properties that can be changed in a measured manner using an external electric field, has become one of the most attractive smart materials. ER fluid is typically a suspension consisting of polarizable particles mixed in a nonpolar liquid medium. When subjected to an external electric field, the ER fluids will alter from a liquid state to a nearly solid state with a growth in the dynamic shear stress and viscosity. Due to dipole–dipole interactions along the direction of the applied electric field, the particles will start to connect to each other in the form of a chain-like structure. Furthermore, the ER fluids will return to their previous state once the external electric field vanishes. The transformation process is extremely rapid, generally less than 10 ms. This exciting characteristic delivers an efficient method for applications, such as shock absorbers, biomedical devices, and robotics.^4–6^ Hence, ER fluid shows hefty available potential in future markets.

Owing to their excellent performance, many ER fluids, consisting of different types of matter, have been developed. In 2014, Choi *et al.*^7^ successfully prepared GO-Si particles with a core–shell structure and the viscosity of the GO-Si-based ER fluid changed by two orders of magnitude under an external electric field. Using the glucose-assisted hydrothermal method, Wang *et al.*^8^ in 2018, made a TiO_2_@MoS_2_-based ER fluid. Then, Hao *et al.*^9,10^ successfully made an MoS_2_@SiO_2_-based ER fluid and an H_2_Ti_2_O_5_@MoS_2_@SiO_2_-based ER fluid in 2021. Kuznetsov *et al.*^11,12^ made a new ER fluid based on chitosan particles in 2021. Meanwhile, Dhar *et al.*^13^ used agarose to make an organic colloidal ER fluid, and Meng *et al.*^14^ successfully made ionic liquid-crystalline polysiloxane-based ER fluids. In 2018 Sedlacik *et al.*^15^ made iron(ii) oxalate particles with a special shape, where the particles had an extremely respectable aspect ratio with their length of ranging from 15 to 30 μm and thickness ranging from 1 to 2 μm.

However, the dynamic shear stress of the numerous ER fluids can be insufficient to support their application,^7,16,17^ and their application is also subject to low applied electric field strength.^18–21^ On the other hand, owing to the difference in density between particle and liquid, the sedimentation ratio, which can indicate the wetting property, also significantly restricts their application.^22–26^ Hence, we have a compelling need to seek a new ER fluid with a higher applied electric field and excellent wetting properties to improve this situation.

In this work, we successfully prepared novel particles by co-precipitation in ethanol and aqueous solution, based on iron(ii) oxalate-doped titanium. We can synthesize them by a simple method without high pressure or high temperature. The corresponding ER fluids, dispersing the particles in silicone oil, show excellent performance compared to previous particles. They have higher dynamic shear stress and can adapt to a stronger electric field intensity so that they can be applied in more scenarios. Furthermore, the time dependence of the viscosity alternating in different electric field strengths is tested. Finally, the sedimentation performance of these ER fluids containing the particles, dried at different temperatures, is verified.

## Experimental

### Materials

Titanium butoxide (TBT) (≥99.0%, Aladdin), iron(ii) chloride tetrahydrate (FeCl_2_·4H_2_O) (AR, 99.0%, Aladdin), oxalic acid dehydrate (H_2_C_2_O_4_·2H_2_O) (AR, ≥99.5%, Aladdin) and *n*-butanol (AR, 99%, Aladdin) were purchased from the Aladdin company (Shanghai, China). Anhydrous ethanol (≥99.5%) was purchased from Sinopharm Group Co. and ultra-pure water (DI) was produced from an ultra-pure water machine (Aquapro). All of the materials cited above were used as received without further purification.

### Synthesis

The particles were made by co-precipitation synthesis. Firstly, 9.94 g FeCl_2_·4H_2_O was dissolved in 200 mL of anhydrous ethanol to form solution A. Meanwhile, 17 mL of TBT was dissolved in 100 mL of anhydrous ethanol to create solution B, and 12.68 g of H_2_C_2_O_4_·2H_2_O was dissolved in 100 mL of DI; then solution C is obtained. After solutions A, B, and C were stirred for 1 h, solution B was added to solution C to create solution D. After mixing, solution D becomes limpid, and clear solution D was added to solution A and was further stirred for 6 h at room temperature of 25 °C. After aging for 8 h, we collected the precipitate and washed it with a large amount of DI and anhydrous ethanol in turn by vacuum suction filtration. Ultimately, the particles were dried at different temperatures, 50 °C, 60 °C, 70 °C, and 80 °C, respectively, for 12 h, and another temperature of 110 °C for 4 h.

### Preparation of ER fluids

Before the experiments, all the silicone oil (*η* = 50 mPa s, 25 °C) was dried at 120 °C for 2 h under vacuum. And the synthetic particles were ground for 12 h in a ball mill. By using a magnetic stirrer and an ultrasonic machine, the ER fluids, containing the particles within silicone oil were obtained with 20 wt% and 5 wt%, respectively.

### Characterization

Using a scanning electron microscope (SEM), the morphology of the particles, sonicated in anhydrous ethanol and dried on a glass slide at 110 °C, was observed. Meanwhile, the chain-like structure of the ER fluids with 2 wt%, mixing the particles in *n*-butyl alcohol, were captured by SEM. The optical image of the chain-like structure in the ER fluids with 5 wt%, containing the particles within silicone oil, was captured using an optical microscope. Then, the Fourier-transform infrared (FTIR) spectra were recorded with a Nicolet iS50. Using a PANalytical X'Pert Powder, the X-ray diffraction (XRD) data was observed by monitoring the diffraction pattern appearing in the 2*θ* angle range of 10–80° with a scan speed of 7° min^−1^.

Using a rotational rheometer (Physica MCR 302, Anton Paar), with a plate–plate geometry (diameter of 25 mm and a gap of 1.0 mm at 25 °C), electrorheological test measurements were performed. The external electric field strengths of 0–3.0 kV mm^−1^ were induced by a high-voltage DC source. To obtain reliable performance, an electric field was applied for 2 min before starting the test in order to provide enough time for the ER fluids to create equilibrium chain-like structures. After testing with an external electric field, a constant shear rate of 300 s^−1^ was applied for 90 s to disrupt the residual structures. The flow curves for the dynamic shear stress *versus* shear rate, which range from 0.1 to 100 s^−1^, were recorded using the rotational stress model. The static yield stress was measured by the rotate-stress model at a very low shear rate (0.1 s^−1^). At the same time, the time dependence of the viscosity alternating between 0 kV mm^−1^ and 3 kV mm^−1^ for the ER fluids was measured at a shear rate of 1 s^−1^. By putting the test tube (5 mL) with the ER fluids on a horizontal test tube rack, data (0–600 h) for the sedimentation ratio were recorded.

## Results and discussion

**Fig. 1:**
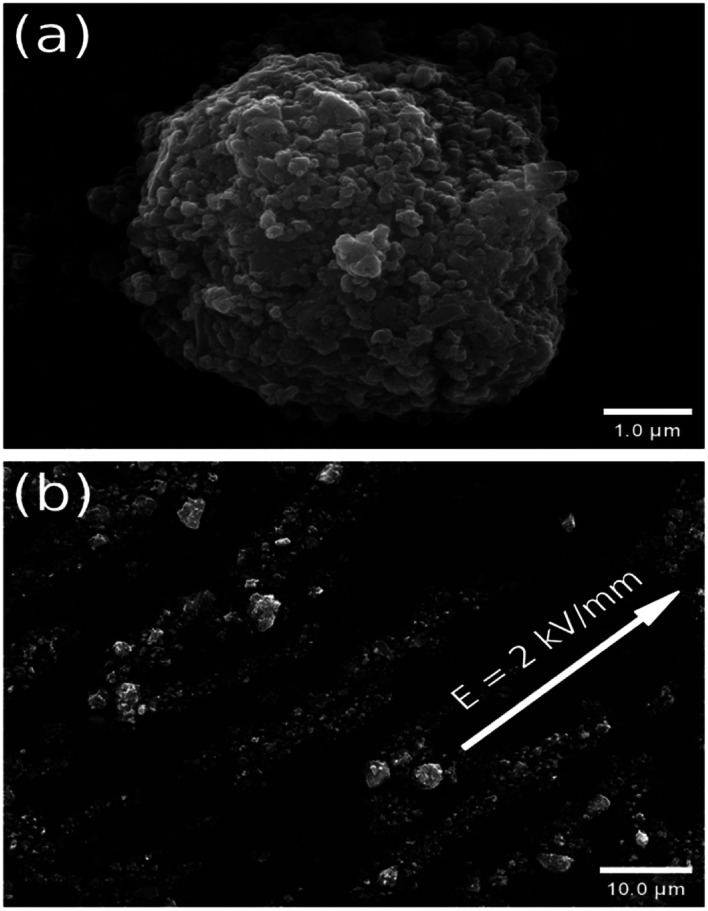
(a) SEM morphology of a particle dried at 70 °C. (b) SEM observation of the chain-like structure in ER fluid with 2 wt% under 2 kV mm^−1^, mixing the particles dried at 80 °C in *n*-butyl alcohol.

The morphology of the ER particles, with the different drying temperatures, was measured by SEM. As can be seen, the morphology of the particles in Fig. 1(a) is more circular than the particles in Fig. S1,† and the respective oval morphology is obtained in Fig. S1(b).† A cauliflower-shaped ball, unlike the morphology of the previous pure iron(ii) oxalate,^15^ is obtained in all the particles, which clearly proves that titanium oxide with a small spherical shape is added to the iron(ii) oxalate.

**Fig. 2:**
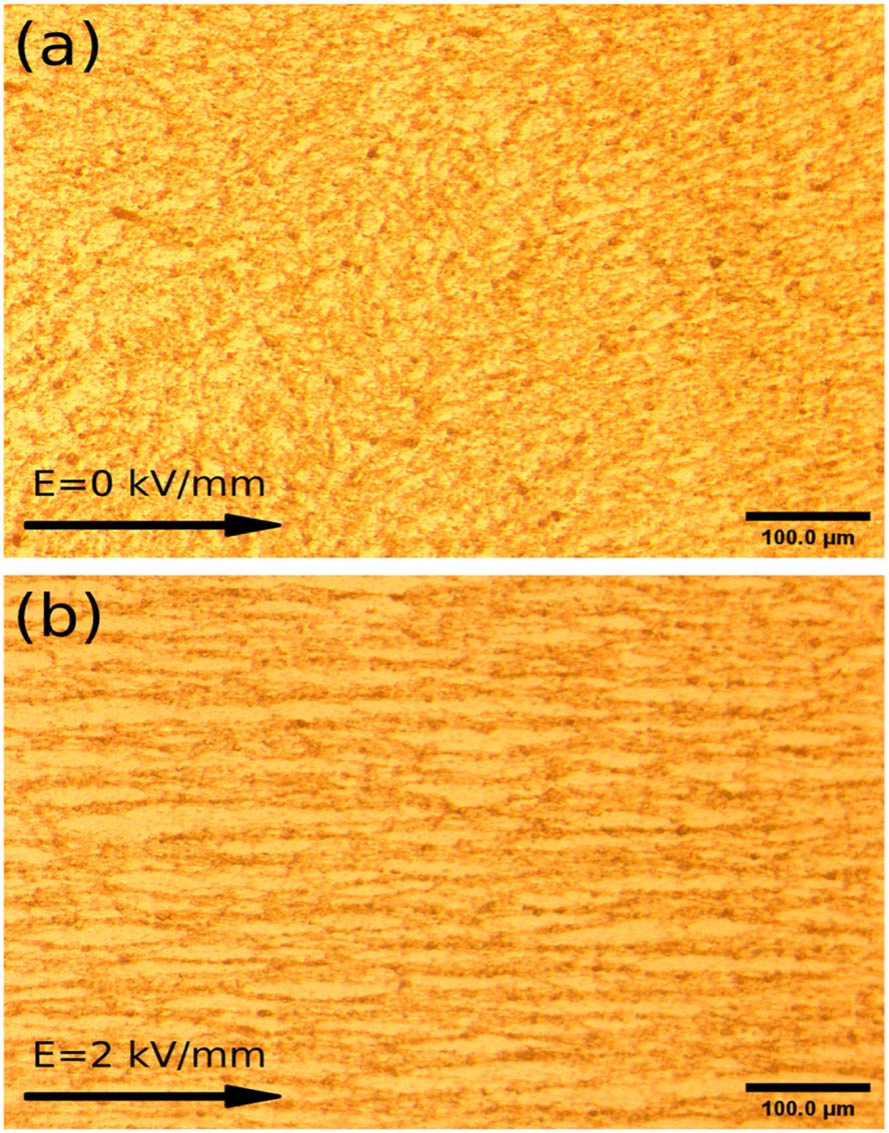
Optical images of ER fluids with 5 wt% mixing of particles dried at 50 °C in silicone oil, (a) without an external electric field and (b) in the presence of a field of 2 kV mm^−1^.

At the same time, thanks to the lower current density, the optical image of the ER fluids with 5 wt% under 2 kV mm^−1^, containing the particles dried at 50 °C within the silicone oil, is given in Fig. 2. In the absence of an external electric field, the image of a disordered fluid is obtained at the beginning. Subsequently, an image of an orderly chain-like structure within the ER fluid is obtained after using an applied electric field of 2 kV mm^−1^ in Fig. 2. As can be seen, the direction of the chain-like structure is obviously coincident with the applied electric field. Meanwhile, fibrous-chain-like structures, within the ER fluids consisting of particles dried at other temperatures, 60 °C, 70 °C, and 80 °C, respectively, are obtained in Fig. S4.† Obviously stronger chain-like structure compared to other ER fluids can be observed in Fig. S4(d).† To compare the differences in the chain-like structures within these ER fluids and take a better look at their microstructural formation, SEM images of the ER fluids with 2 wt% under 2 kV mm^−1^, containing the particles within *n*-butyl alcohol, were obtained, as shown in Fig. 1(b) and S2.† As can be seen, the direction of the fibrous-chain-like structure within particles is parallel to the external electric field, because the particles are polarized along the direction of the electric field.

The EDS spectra of the particles dried at different temperatures are shown in Fig. S3.† It can be clearly seen that the iron(ii) oxalate is successfully doped with titanium and the concentration of titanium is evidently affected by the drying temperature, so there is an obvious difference in the rheological properties. Furthermore, the lowest concentration of titanium was obtained in the particles dried at 70 °C and the highest was obtained at 50 °C. The corresponding rheological properties will be discussed in detail in Fig. 4 and S5.†

**Fig. 3:**
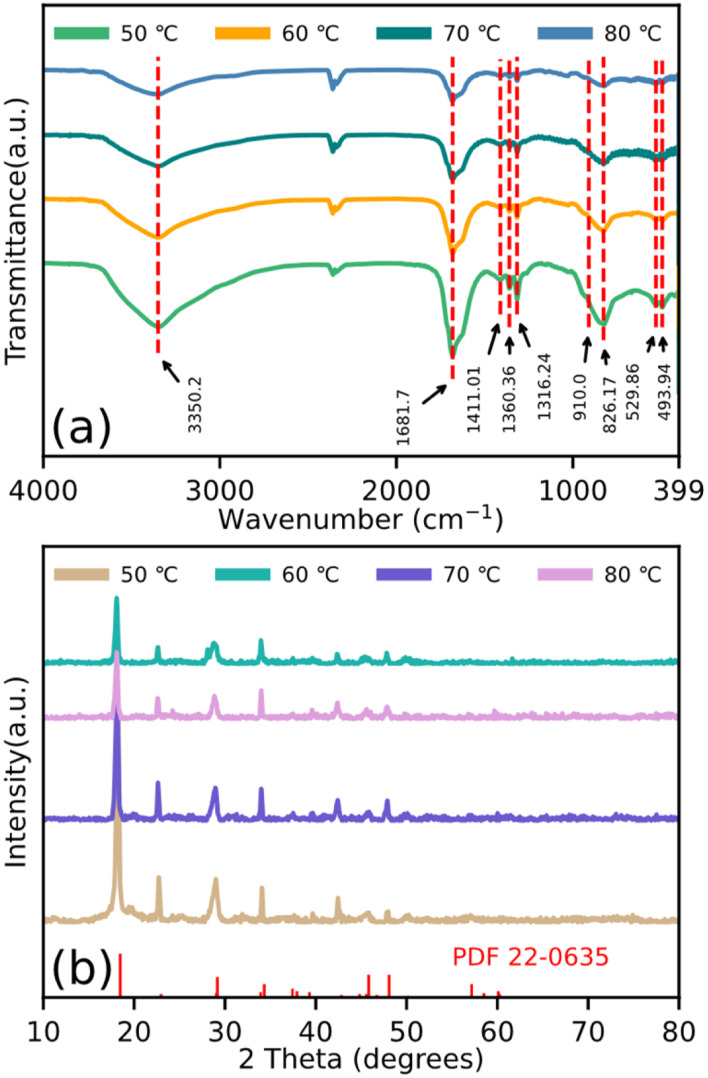
(a) FTIR spectroscopy and (b) XRD results of particles dried at temperatures of 50 °C, 60 °C, 70 °C, and 80 °C.

The FTIR spectra (Fig. 3(a)) were also measured to verify the formation of these particles. For the existing stretching vibration of the –OH group, the broad band at approximately 3350.2 cm^−1^ is shown. The peak at 1681.7 cm^−1^ is attributed to the O–H in absorbed water. The bands at 1411.01, 1360.36, and 1316.24 cm^−1^ are attributed to metal carboxylate (M–COO–) symmetric stretching.^27,28^ The band at 910.0 cm^−1^ is attributed to the Ti=O band, 826.17 cm^−1^ is because of the C–C vibration mode, and 529.86 cm^−1^ is assigned to the C–O in-plane bending. In addition, the band at 493.94 cm^−1^ is attributed to the Fe–O stretching vibration. The XRD data is shown in Fig. 3(b). It can be obviously seen that the structures within the particles are similar to each other. Furthermore, in contrast to the reported X-ray data for β-iron(ii) oxalate (PDF 22-0635), the diffraction peak with the low angle movement in these particles is also shown.

**Fig. 4:**
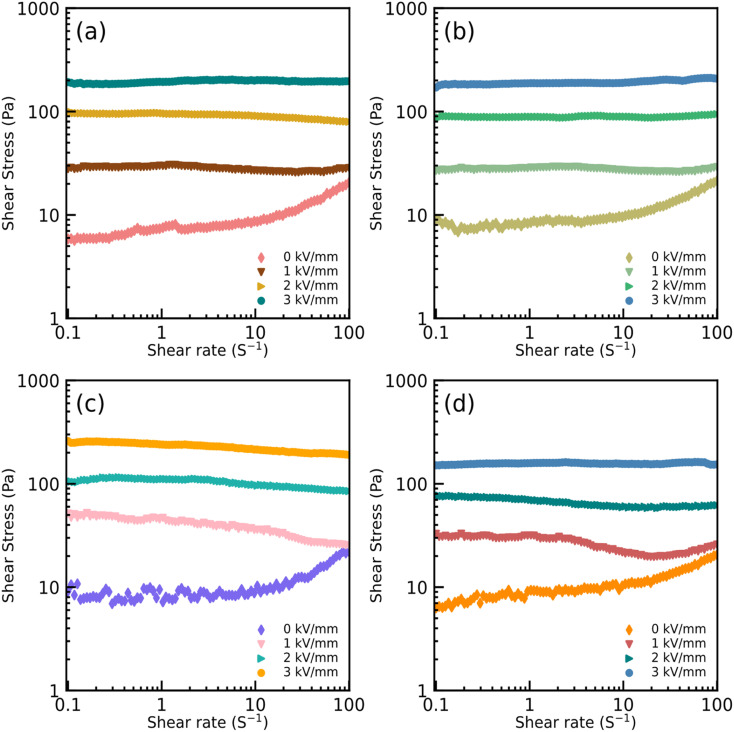
Dependence of dynamic shear stress on the shear rate for ER fluids with 20 wt%, dispersing the particles dried at different temperatures ((a) 50 °C, (b) 60 °C, (c) 70 °C, and (d) 80 °C) in silicone oil, under various electric fields (0–3 kV mm^−1^).

Fig. 4 and S5† show the rheological properties of the ER fluids containing particles dried at different temperatures. All the fluids formed a chain-like structure under an electric field intensity of 3 kV mm^−1^, which proves that these ER fluids exhibit better insulation and lower current density than in previous work.^15^ As can be seen, the dynamic shear stress of these ER fluids shows a linear increase along with the dependence of the shear rate without an applied electric field, showing almost Newtonian behavior. However, with an increase in electric field intensity, the dynamic shear stress of all the ER fluids is synchronously increasing. Thanks to the electrostatic interaction within the particles under the external electric field, a relatively flat area appears, which shows the fibrous-chain-like structure has appeared within the dispersed particles under the applied electric field. This effect also leads to an increase in the viscosity of the system. Due to the better insulating properties and the lower current density than previous particles, the fibrous-chain-like structure within these ER fluids becomes more stable after applying a stronger electric field. At the same time, owing to the stouter chain-like structure obtained under the higher electric field, the corresponding dynamic shear stress and viscosity increase with dependence on the external electric field in Fig. 4 and S5,† respectively. Under an electric field intensity of 3 kV mm^−1^, the strength of the dynamic shear stress in Fig. 4(c) was higher than the others shown in Fig. 4, while the curve of the dynamic shear stress was not as flat as the others. This result confirms that the chain-like structure in Fig. 4(c) is not as dense as others, but the structure is coarser than others, and this can also be proved by the obvious difference between Fig. 2 and S4.† Furthermore, Fig. S5† also shows that excellent rheological properties are observed for these ER fluids, based on which the dependence of their viscosity on the shear rate can be obtained. As can be seen, the viscosity decreases gradually with an increase in the shear rate, and the maximum value of the viscosity is obtained in Fig. S5(c)† for the changes within the structures at an invariant shear rate. All the ER fluids, thanks to the lower current density, can work well at a higher electric field intensity, and the enhancement in the viscosity is attributed to their obvious chain-like structure. Therefore, the advantage of the ER fluids presented in this work can be marked by the reasonably good ER properties compared to previous work.^15^

The ER efficiency, *e* (eqn (1)), is a very important property for ER fluids. Some previous ER fluids cannot achieve a satisfactory value,^29–31^ one of the reasons for which is the unsatisfactory rheological properties. In this work, the efficiency can be calculated as:1
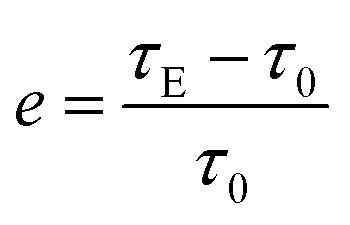
where *τ*_E_ is the static yield stress in the presence of the external electric field, and *τ*_0_ is the field-off static yield stress.

**Fig. 5:**
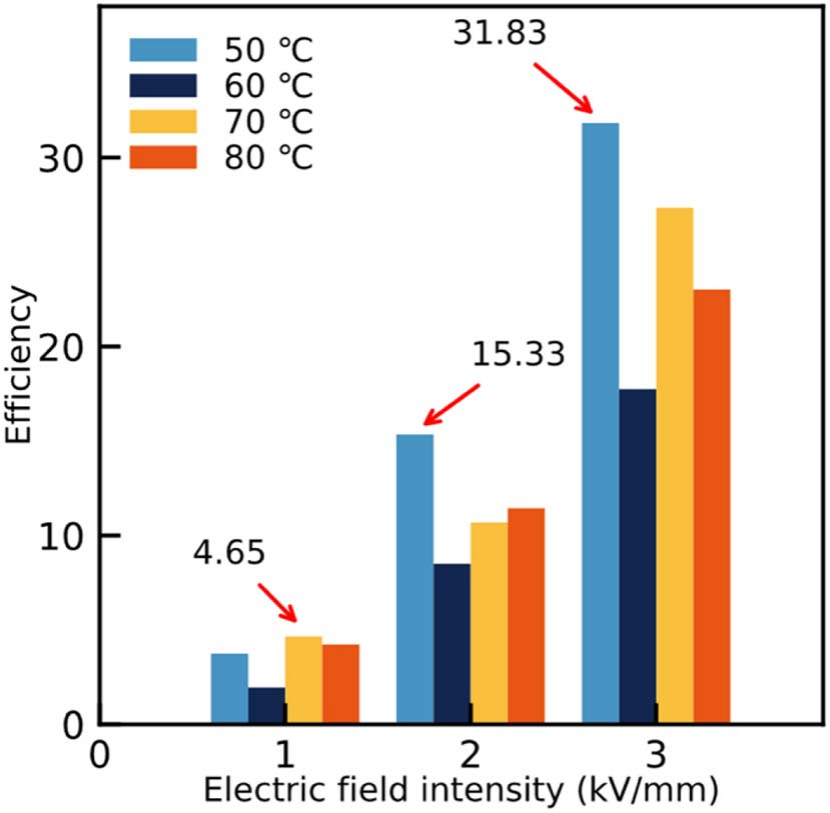
The ER efficiencies of the different ER fluids with 20 wt%, mixing the particles dried at different temperatures (50 °C, 60 °C, 70 °C, and 80 °C) in silicone oil, under different external electric field strengths (1 kV mm^−1^, 2 kV mm^−1^, and 3 kV mm^−1^). The maximum efficiencies were 4.65, 15.33, and 31.83, respectively.

Fig. 5 shows that the ER efficiencies of the ER fluids are obviously different. Thanks to the appearance of the chain-like structure for ER fluids consisting of identical particles, the corresponding ER efficiency increases following the electric field intensity. At the same time, owing to the subtle differences in the chain-like structure, the values of the ER efficiency also show differences under the same electric field intensity. The value can reach 4.65 in ER fluids consisting of particles dried at 70 °C under 1 kV mm^−1^, while for those containing particles dried at 50 °C, the values could achieve 15.33 and 31.83 under 2 kV mm^−1^ and 3 kV mm^−1^, respectively. Compared to previous research,^31^ the ER fluids in our work show excellent ER efficiency.

**Fig. 6:**
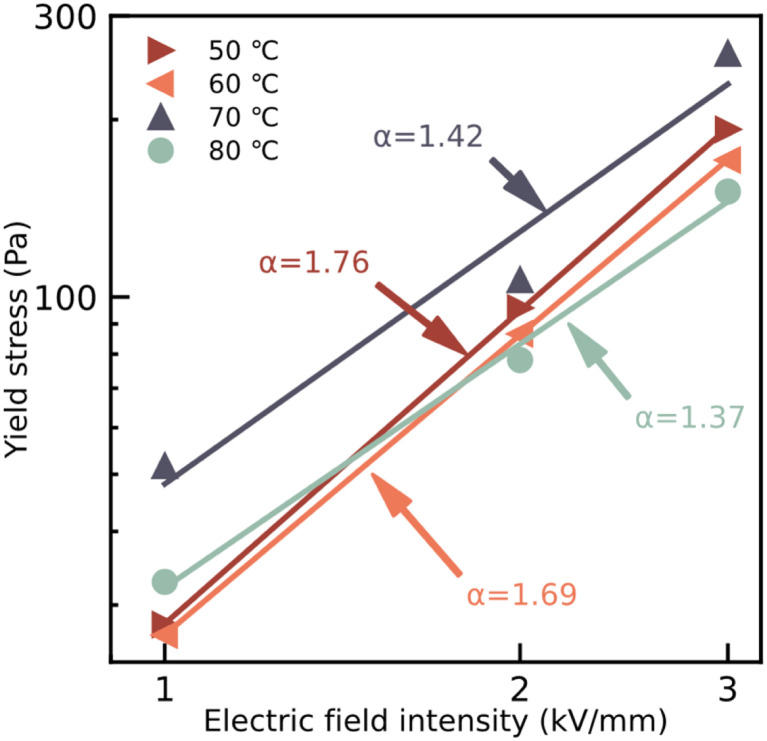
Dependence of static yield stress on applied electric field intensity for tER fluids with 20 wt%, mixing the particles dried at temperatures of 50 °C, 60 °C 70 °C, and 80 °C, in silicone oil.

Fig. 6 shows the relationship between the static yield stress of the ER fluids and the external electric field, which is obtained from the data on dynamic shear stress with a shear rate of 0.1 s^−1^. The regulatory effect of the static yield stress of the ER fluids under the applied electric field is shown as expected. The relationship between the static yield stress and the external electric field intensity follows:

2 *τ*_y_ = *q* × *E*_c_*α*

where *τ*_y_ represents the static yield stress of the ER fluids, the rigidity of the system is expressed by the parameter *q*, *E*_c_ indicates the applied electric field strength and *α* is the exponential factor fitting the data. Generally, the model formed of the ER fluids is always determined by the value of *α*. For example, the internal chain-like structure in the ER fluids is fitted by the conductive model if *α* is 1.5, and when *α* is 2, it is led by the molecular polarization model.^15,32^ In Fig. 6, the values of *α* of ER fluids based on particles dried at temperatures of 50 °C, 60 °C, 70 °C, and 80 °C are obtained, and the corresponding values are close to 1.76, 1.69, 1.42, and 1.37, respectively. The results mean the conductive model played a major role in these ER fluids.

**Fig. 7:**
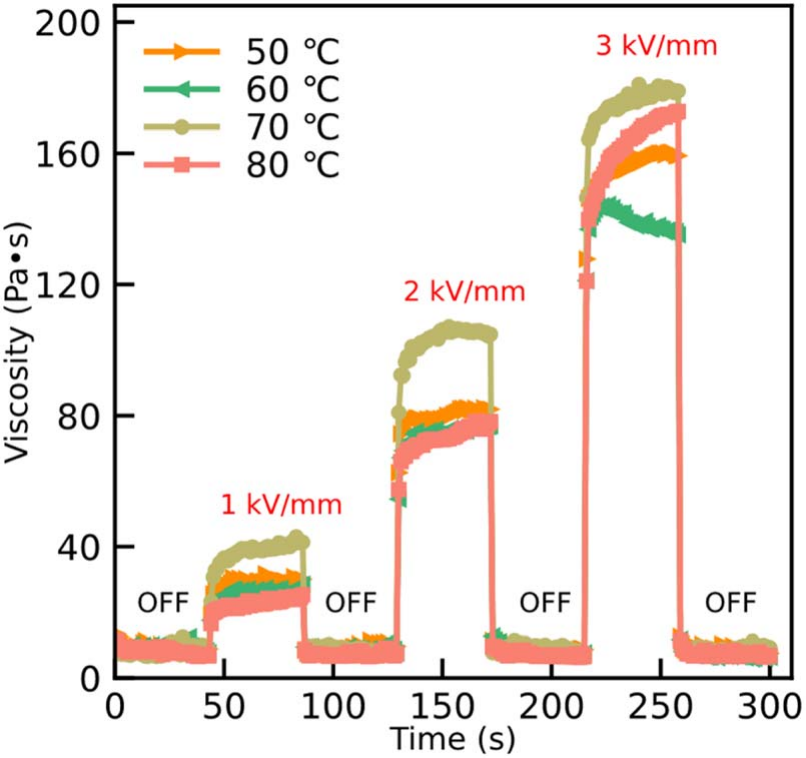
Time dependence of viscosity alternating between 0 kV mm^−1^ and 3 kV mm^−1^ at ta shear rate of 1 s^−1^ for ER fluids with 20 wt%, dispersing the particles dried at temperatures of 50 °C, 60 °C, 70 °C, and 80 °C, in silicone oil.

According to the time process of viscosity at a shear rate 1 s^−1^, Fig. 7 shows the ER reproducibility after switching on and off the external electric field. Without applying the external electric field, the viscosity is comparatively rapidly returned to its initial value owing to the disappearing internal chain-like structure. However, the internal chain-like structure in the ER fluids is established by applying an external electric field and this effect leads the value of the viscosity to change accordingly; see Fig. 7. The results show that the response time of the particles, with and without an external electric field, is quick and the electric field can be adjusted over a large range owing to the lower current density in this work.

**Fig. 8:**
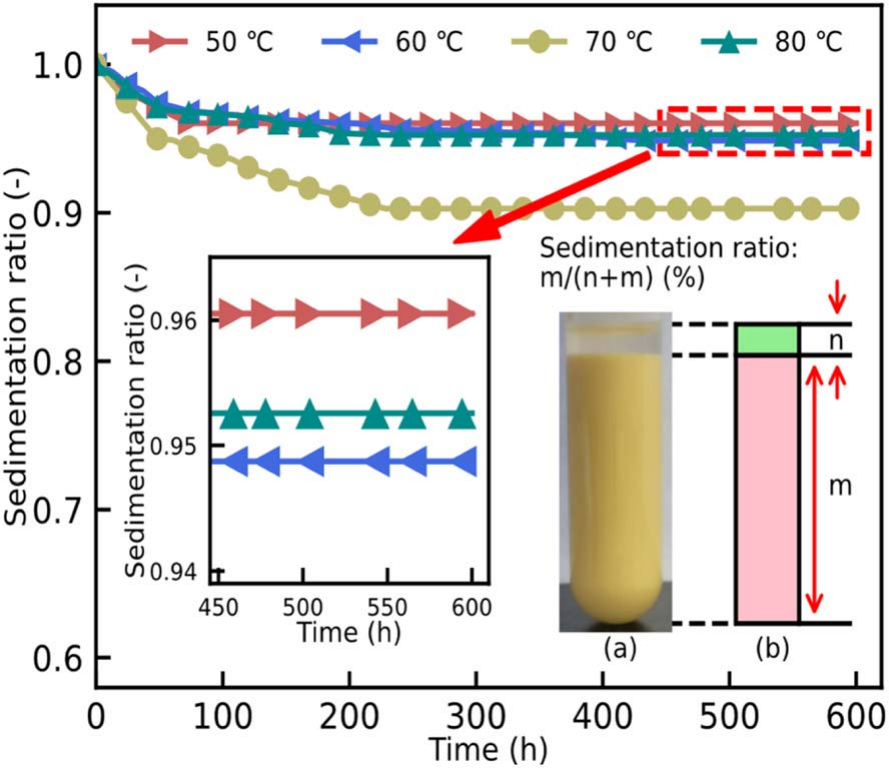
The sedimentation performance of ER fluids with 20 wt%, mixing these particles dried at temperatures of 50 °C, 60 °C, 70 °C, and 80 °C, in silicone oil, from 0 h to 600 h. (a) The ER fluid containing particles dried at 70 °C. (b) A schematic diagram of the sedimentation rate calculation, where *n* and *m* represent the clear liquid height and the particle sedimentation layer height, respectively.

The other important factor restricting the development of ER fluids is the sedimentation ratio, which can show wetting properties between the particles and the liquid medium in the ER fluids. The calculated values of the ratio are shown in Fig. 8. Owing to the difference in the density between the particles and the liquid medium, the appearance of the phenomenon of phase separation leads to an unsatisfactory ER property. Fig. 8 shows the excellent result of the sedimentation ratio from 0 h to 600 h. As can be seen, the sedimentation ratios of all these ER fluids show a rapid descent in the first 300 h, after which invariant ratios are obtained. Although the dynamic shear stress of the ER fluids, mixed with particles dried at 70 °C, shows an excellent result, its sedimentation ratio is obviously inferior to others in Fig. 8. At the same time, the best performance is shown in the ER fluid consisting of particles dried at 50 °C. Its sedimentation ratio could be above 0.96. Compared to previous reports,^15,23^ the sedimentation ratio of the ER fluids in this work shows an advantageous result.

## Conclusions

In this work, iron(ii) oxalate doped titanium particles, with the morphology of a cauliflower-shaped ball, were successfully obtained *via* a simple method of co-precipitation in ethanol and aqueous solution. The EDS results show that the iron(ii) oxalate successfully doped the titanium and the molecular structure was proved by XRD and FTIR spectroscopy. After applying an external electric field, an obvious fibrous-chain-like structure within the ER fluids consisting of particles dried at different temperatures was observed by SEM and optical microscopy. Owing to the excellent insulation and lower current density, the rheological properties of these ER fluids were tested under different electric fields (0–3 kV mm^−1^). The results show advantageous rheological properties in contrast to ER fluids of pure iron(ii) oxalate. Furthermore, the highest ER efficiency in this work was found in the ER fluid consisting of particles dried at 50 °C under 3 kV mm^−1^. Finally, the sedimentation ratio of these ER fluids was obtained from 0 h to 600 h, indicating that all the ER fluids in this work have excellent wetting properties. To sum up, the enhancement of the ER behavior in iron(ii) oxalate doped titanium was confirmed with certainty in this work.

## Conflicts of interest

There are no conflicts to declare.

## Supplementary Material

RA-012-D2RA05608A-s001

## References

[cit1] Dong Y. Z., Seo Y., Choi H. J. (2019). Soft Matter.

[cit2] Cheng H., Lim T., Kim S., Jung W. (2022). Mater. Res. Lett..

[cit3] Liang Y., Huang D., Zhou X., Wang Z., Shi Q., Hong Y., Pu H., Zhang M., Wu J., Wen W. (2022). Engineering.

[cit4] Wang R., Wang Y. C., Feng C. Q., Zhou F. (2012). Adv. Mater. Res..

[cit5] Kamelreiter M., Kemmetmüller W., Kugi A. (2012). Mechatron..

[cit6] Koyanagi K., Takata Y., Kakinuma Y., Anzai H., Sakurai K., Motoyoshi T., Oshima T. (2013). J. Phys.: Conf. Ser..

[cit7] Kim S. D., Zhang W. L., Choi H. J., Seo Y. P., Seo Y. (2014). RSC Adv..

[cit8] He K., Wen Q., Wang C., Wang B., Yu S., Hao C., Chen K. (2018). Chem. Eng. J..

[cit9] Chen Y., Sun W., Zheng H., Li C., Zhang B., Wang B., Hao C. (2021). Ceram. Int..

[cit10] Sun W., Xi Z., Zheng H., Chen Y., Li C., Wang B., Hao C. (2021). J. Taiwan Inst. Chem. Eng..

[cit11] Kuznetsov N. M., Zagoskin Y. D., Vdovichenko A. Y., Bakirov A. V., Kamyshinsky R. A., Istomina A. P., Grigoriev T. E., Chvalun S. N. (2021). Carbohydr. Polym..

[cit12] Kuznetsov N. M., Zagoskin Y. D., Bakirov A. V., Vdovichenko A. Y., Malakhov S. N., Istomina A. P., Chvalun S. N. (2021). ACS Sustainable Chem. Eng..

[cit13] Dhar P., Saini V., Chattopadhyay A., Samanta D. (2021). Phys. Fluids.

[cit14] Li X., Chang X., Zheng X., Kong W., Zhuang Y., Yan G., Meng F. (2021). Eur. Polym. J..

[cit15] Kutalkova E., Plachy T., Osicka J., Cvek M., Mrlik M., Sedlacik M. (2018). RSC Adv..

[cit16] Kim Y. D., Hong G. G. (2012). Korean J. Chem. Eng..

[cit17] Sedlačík M., Mrlík M., Pavlínek V., Sáha P., Quadrat O. (2011). Colloid Polym. Sci..

[cit18] Liu Y., Choi H. (2013). Chem. Pap..

[cit19] Fang F. F., Liu Y. D., Choi H. J. (2013). Colloid Polym. Sci..

[cit20] Marins J. A., Soares B. G., Silva A. A., Hurtado M. G., Livi S. (2013). J. Colloid Interface Sci..

[cit21] Wang Z., Song X., Wang B., Tian X., Hao C., Chen K. (2014). Chem. Eng. J..

[cit22] Egorysheva A. V., Kraev A. S., Gajtko O. M., Kusova T. V., Baranchikov A. E., Agafonov A. V., Ivanov V. K. (2020). Powder Technol..

[cit23] Yoon C. M., Ryu J., Yun J., Kim Y. K., Jang J. (2018). ACS Appl. Mater. Interfaces.

[cit24] Wang J., Chen G., Yin J., Luo C., Zhao X. (2017). Smart Mater. Struct..

[cit25] Ma N., Niu C., Dong X., Han B. (2017). Mater. Res. Express.

[cit26] He K., Wen Q., Wang C., Wang B., Yu S., Hao C., Chen K. (2017). Soft Matter.

[cit27] Wu J., Zhang L., Xin X., Zhang Y., Wang H., Sun A., Cheng Y., Chen X., Xu G. (2018). ACS Appl. Mater. Interfaces.

[cit28] Wu J., Song Z., Liu F., Guo J., Cheng Y., Ma S., Xu G. (2016). NPG Asia Mater..

[cit29] Omambala J. R., McIntyre E. C., Gallo A. A. (2019). ACS Omega.

[cit30] Danilin A., Kydralieva K., Semenov N., Kelbysheva E. (2021). Mater. Today: Proc..

[cit31] Brehm T., Pereira G., Leal C. R., Gonçalves C., Borges J. P., Cidade M. T. (2015). Phys. Scr..

[cit32] Zhang K., Liu Y. D., Jhon M. S., Choi H. J. (2013). J. Colloid Interface Sci..

