# Supplementary material for: Enhanced response of titanium doped iron(ii) oxalate under electric field
Source: RSC Adv. 2022 Nov 8;12(49):31959–65. doi: 10.1039/d2ra05608a (PMC9641721; doi:10.1039/d2ra05608a)
Supplement: RA-012-D2RA05608A-s001 [file RA-012-D2RA05608A-s001.pdf]

Electronic Supplementary Material (ESI) for RSC Advances.
This journal is © The Royal Society of Chemistry 2022

Enhanced response of titanium doped iron(II) oxalate under electric field

Chunde Li, [a] Hua Wei, [*a,b] Xueyan Hu, [a] Zhaoxian Chen, [a] Xin Xie, [a] Guo Chen, [a] Anping Liu, [a] Yingzhou Huang, [a] and Weijia Wen [c]

[a] *Chongqing Key Laboratory of Soft Condensed Matter Physics and Smart Materials, College of Physics, Chongqing University, Chongqing 401331, China. Email: huawei.hw@cqu.edu.cn*

[b] *State Key Laboratory of Mechanical Transmissions, Chongqing University, Chongqing 400044, China.*

[c] *Department of Physics, The Hong Kong University of Science and Technology, Clear Water Bay, Kowloon, Hong Kong, China.*

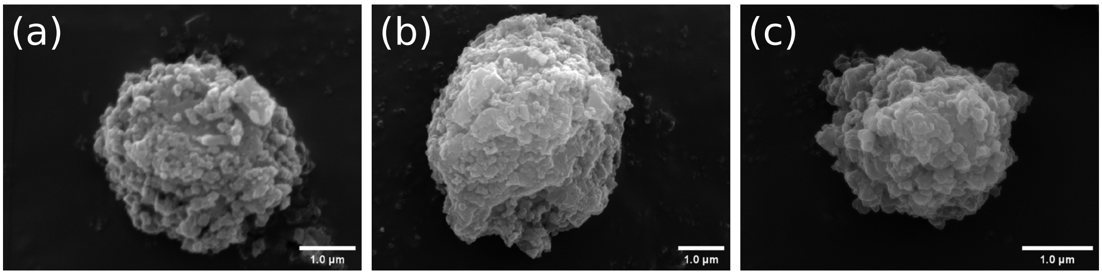

Fig S1: The SEM morphology of the particle dried at temperatures, **(a)** 50 ℃, **(b)** 60 ℃, and **(c)** 80 ℃, respectively.

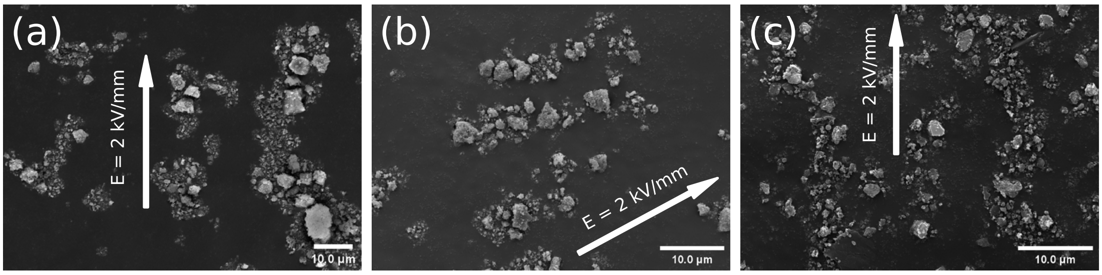

Fig S2: The SEM observation of the chain-like structure in the ER fluids with 2 wt %, mixing the particles dried at temperatures, **(a)** 50 ℃, **(b)** 60 ℃, and **(c)** 70 ℃, respectively, in the N-butyl alcohol, under 2 kV/mm.

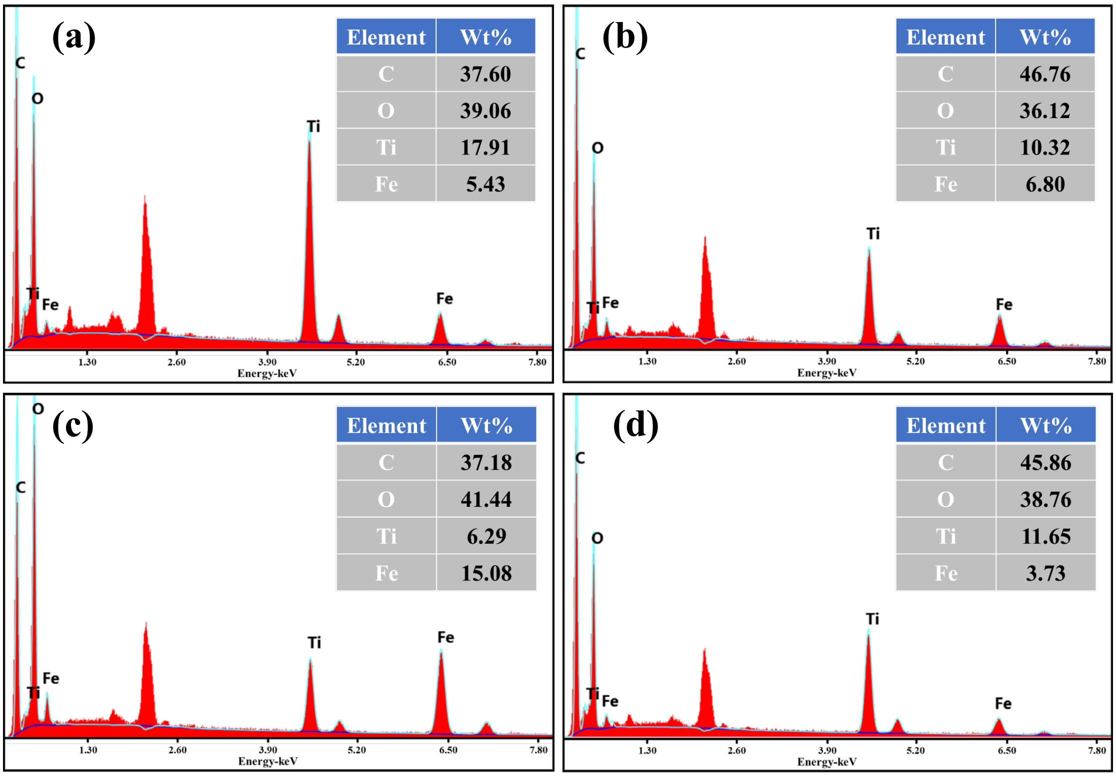

Fig S3: The EDS spectra of the particles dried at temperatures, **(a)** 50 ℃, **(b)** 60 ℃, **(c)** 70 ℃, and **(d)** 80 ℃, respectively.

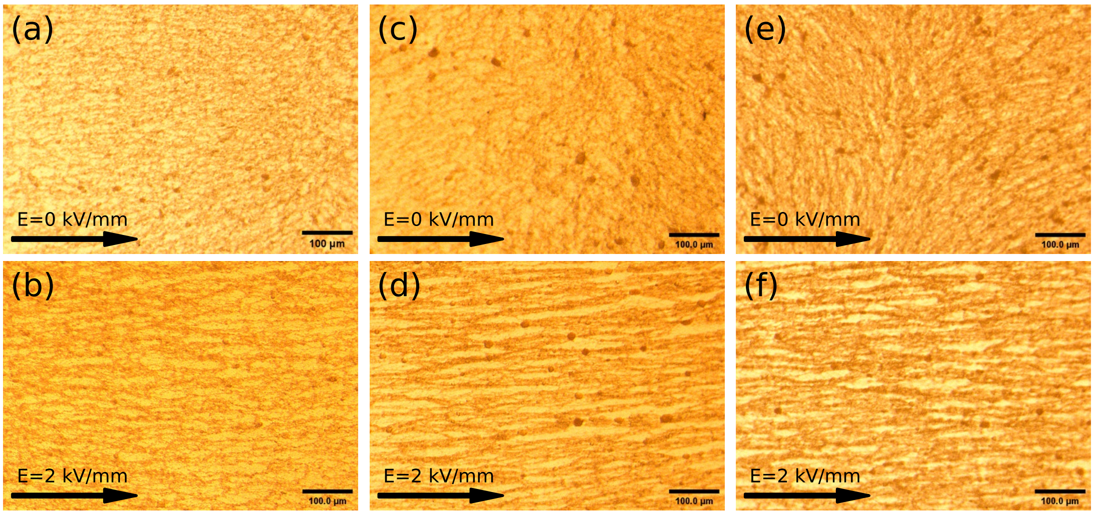

Fig S4: The optical image of the ER fluids with 5 wt %, consisting the particles dried at temperatures, **(a)(b)** 60 ℃, **(c)(d)** 70 ℃, and **(e)(f)** 80 ℃, respectively, in silicone oil, under defecting the external electric field **(a) (c) (e)**and existing the field **(b) (d) (f)** with 2 kV/mm.

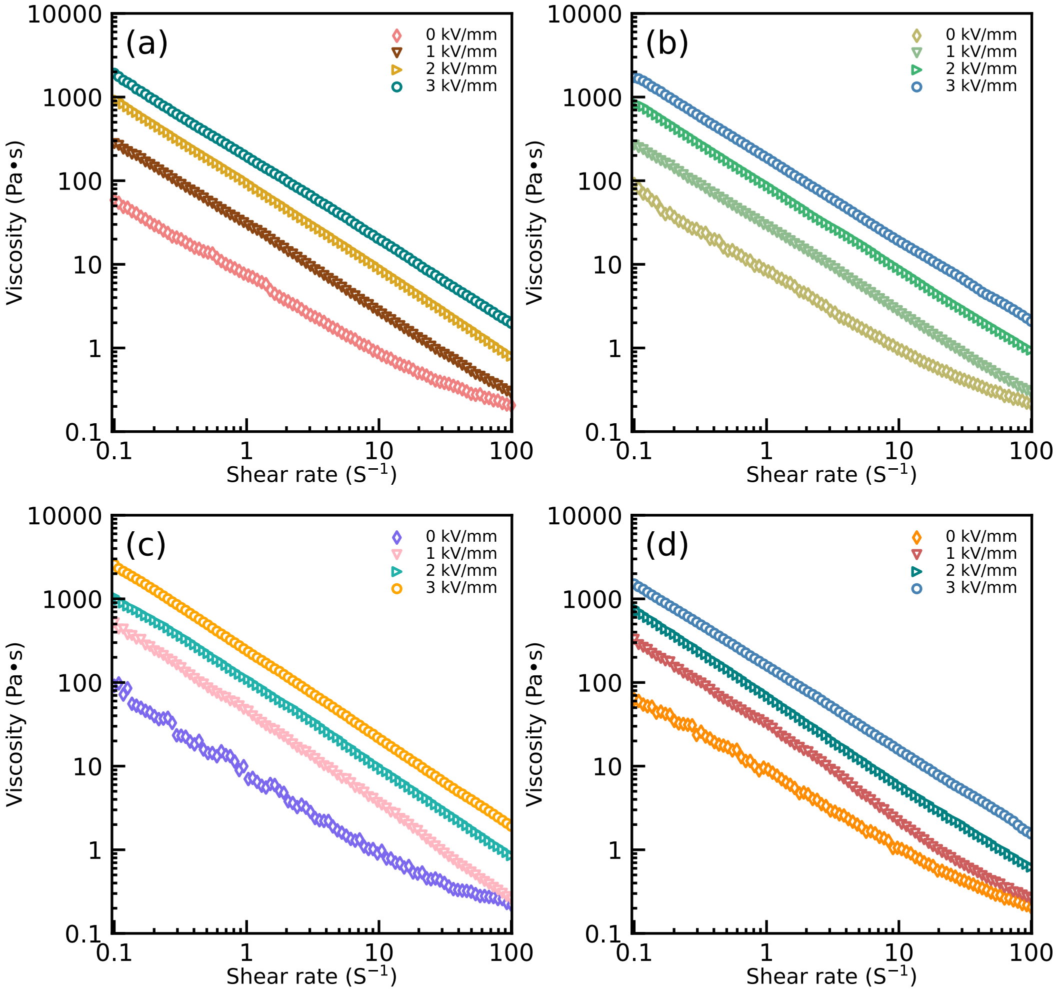

Fig S5: Dependences of the viscosity on the shear rate for the ER fluids with 20 wt %, mixing the different particles dried at temperatures, **(a)** 50 °C, **(b)** 60 °C, **(c)** 70°C, and **(d)** 80 °C, respectively, in silicone oil, under the various electric fields (0 – 3 kV/mm).
